# Greater Reliance on Cerebral Palsy-Specific Muscle Synergies During Gait Relates to Poorer Temporal-Spatial Performance Measures

**DOI:** 10.3389/fphys.2021.630627

**Published:** 2021-02-23

**Authors:** Yushin Kim, Thomas C. Bulea, Diane L. Damiano

**Affiliations:** ^1^Major of Sports Health Rehabilitation, Cheongju University, Cheongju, South Korea; ^2^Functional and Applied Biomechanics Section, Rehabilitation Medicine Department, National Institutes of Health, Bethesda, MD, United States

**Keywords:** muscle synergy, cerebral palsy, coordination, gait, electromyography, motor module

## Abstract

Children with cerebral palsy typically exhibit reduced complexity of muscle coordination patterns during walking; however, the specific patterns that characterize their gait abnormalities are still not well documented. This study aimed to identify the specific repertoire of muscle coordination patterns in children with CP during walking compared to same-aged peers without CP and their relationships to gait performance. To identify muscle coordination patterns, we extracted muscle synergies from 10 children with CP and 10 age-matched typically developing children (TD). K-mean clustering and discriminant analyses of all extracted synergies were used to group similar synergies. Then, weight-averaged z-scores were quantified for each cluster to determine their group-specific level. In this cohort, 10 of the 17 distinct clusters were largely CP-specific while six clusters were seen mainly in TD, and one was non-specific. CP-specific clusters generally showed merging of two TD synergies, excessive antagonist co-activation, decreased muscle activation compared to TD, and complex or atypical pattern. Significant correlations were found between weight-averaged z-scores and step length asymmetry, cadence asymmetry, self-selected treadmill speed and AP-COM displacement of the pelvis such that greater CP-specificity of muscle synergies was related to poorer performance, thus indicating that CP-specific synergies can influence motor dysfunction.

## Introduction

Walking is an essential daily activity for many children with cerebral palsy (CP). Their specific gait patterns are attributable to multiple factors such as weakness, spasticity, poor selective activation of muscles, abnormal spinal reflexes, and musculoskeletal changes (Damiano et al., 1995; Crenna, 1998). Furthermore, it was postulated decades ago that altered projections from the motor cortex to spinal motor neurons may contribute to the specific gait patterns in children with CP (Brouwer and Ashby, 1991).

Research incorporating electromyography (EMG) analysis during walking in children with CP has shown alteration of muscle activation patterns such as reduced EMG amplitude, agonist-antagonist muscle co-activation, and altered activation timing (Berger et al., 1982; Crenna, 1998; Leonard et al., 2008). These altered patterns are considered a consequence of the disruption in brain development and maturation by the hallmark impairment in supraspinal control (Berger, 1998). Likewise, EMG analyses indicate that children with CP have unique gait strategies with specific muscle activity patterns that typically developed children are unable to mimic (Romkes and Brunner, 2007).

To better illustrate specific EMG patterns in individuals with brain disorders such as stroke and CP, muscle synergies have been analyzed using the non-negative matrix factorization algorithm (Bowden et al., 2010; Rodriguez et al., 2013; Kim et al., 2018; Steele et al., 2019). In neuromechanics, muscle synergies have been hypothesized as representing motor commands of the central nervous system (Ting et al., 2015). Previous studies on muscle synergies identified coordinated patterns of multi-segmental muscles consistently activating together to achieve a task (Ivanenko et al., 2003, 2004, 2005; McGowan et al., 2010; Torres-Oviedo and Ting, 2010; Ting et al., 2015). Specifically, previous studies proposed that the muscle synergies for walking are related to control signals of spinal pattern generators (Ivanenko et al., 2003) and that the superposition of muscle synergy patterns may be related with human compound kinematic (Ivanenko et al., 2005) or kinetic demands (McGowan et al., 2010). A study in healthy populations reported that the basic muscle synergies and overall kinematics rarely vary despite changes in gait speed or the level of body weight support but there is evidence that the synergies are connected to the kinematic walking patterns themselves (Ivanenko et al., 2004). This is also confirmed by the addition of a new synergy if an extra voluntary movement, such as kicking, is included in the walking (Ivanenko et al., 2005). Previous studies also demonstrated that treadmill locomotion shares similar muscle synergy structures with overground locomotion (Oliveira et al., 2016; Mileti et al., 2020). Similarly, balance-specific muscle synergies were consistently recruited in different types of postural tasks (Torres-Oviedo and Ting, 2010). In this context, a review study proposed muscle synergy analysis as a clinical tool for assessing sensorimotor control deficits specific to an individual and defining targets for the rational development of novel rehabilitation therapies (Ting et al., 2015). Thus, the assumption that different synergy structures will be identified that could provide greater insights into altered motor command of the central nervous system and atypical gait kinematics or poorer gait performance in children with CP is reasonable but not yet confirmed.

Clinical studies found that analyzing muscle synergies is useful to reveal the characteristics of muscle coordination patterns of individuals with brain injury (Cheung et al., 2012) and to assess motor function (Bowden et al., 2010; Steele et al., 2015; Shuman et al., 2018). Specifically, the number of synergies deployed during movement has demonstrated utility for quantifying the complexity of descending motor commands from the central nervous system (Bowden et al., 2010; Clark et al., 2010). In children with CP, a reduced number of muscle synergies during walking has been found when compared to typically developed controls, indicating lower motor complexity (Steele et al., 2015; Kim et al., 2018).

In addition to the number of synergies deployed, other parameters calculated from muscle synergy analyses have emerged such as variability and synergy structure composition (weight or vector values that quantify individual muscle contributions to synergies). It was demonstrated that greater variability of muscle synergy structures is associated with poorer motor performance in clinical populations (Allen et al., 2017; Kim et al., 2018). Specifically, it has been shown that children with CP have greater stride-to-stride variability of muscle synergy structures during walking than those in age-matched controls (Kim et al., 2018). Also, the same study demonstrated that some CP-specific muscle synergies are not present in typically developing children. Identifying CP-specific muscle synergies at the level of the individual with CP may provide useful information to establish more individually tailored rehabilitation strategies; e.g., if a child with CP demonstrates two different synergies across strides for the same gait phase with one being more similar to those with typical development, differences in muscle weightings or activation patterns could provide insights on which muscles should be trained or inhibited to shift them toward a more effective pattern of activation.

While the methodology utilized in that initial study is promising, results were preliminary (Kim et al., 2018). The aim here is conduct a larger study incorporating more muscles and gait cycles to more fully identify the repertoire of muscle coordination patterns in children with unilateral CP and to potentially reveal more CP specific muscle synergies during gait. Since current literature on gait reports that children with CP have different muscle synergies (Steele et al., 2015; Kim et al., 2018) and EMG activation patterns (Berger et al., 1982; Crenna, 1998; Leonard et al., 2008), we primarily hypothesized that children with CP would exhibit some specific muscle synergies rarely if at all shown in those with typical development. Based on our previous work (Kim et al., 2018), we also expected to identify non-specific, or common, muscle synergies between children with CP and healthy controls as well as some that were only seen in those without CP. Also, we aimed here to investigate the effect of CP-specific muscle synergies on gait performance. Previously, children with CP have shown decreased walking speed and increased center of mass (COM) displacement during postural control and walking tasks, and 3D kinematic differences as quantified by the Gait Deviation Index (Schwartz and Rozumalski, 2008) when compared with healthy controls (Prosser et al., 2010; Damiano et al., 2013; Zollinger et al., 2016). Given that the timing of muscle synergy activation is correlated with that of specific biomechanical gait events (Lacquaniti et al., 2012), it was hypothesized that walking performance would be correlated with relative weighting of the CP-specific muscle synergies, such that greater CP-specificity in synergy structures would relate to poorer performance.

## Materials and Methods

### Participants

Participants included 10 children with unilateral CP (age, 14.9 ± 3.8 year; body mass, 57.0 ± 20.6 kg; height, 160.4 ± 17.6 cm; 6 right dominant legs; 4 left dominant legs) and 10 children with typical development (TD) within the same age range (age, 15.0 ± 3.2 year; body mass, 69.9 ± 22.1 kg; height, 164.4 ± 16.0 cm; 10 right dominant legs). Seven females were included in each group. Six children with CP were classified as Gross Motor Function Classification System (GMFCS) level I and four as level II. Sample size was calculated based on a previous muscle synergy study (Kim et al., 2018). We calculated sample size using the number of muscle synergies. Considering the means and standard deviations of the variables between children with CP and TD, the sample size needed was 10 subjects in each group. Exclusion criteria were botulinum toxin injections within the previous 4 months, orthopedic surgery to the legs, or a seizure within the previous 6 months. The institutional review board approved the study protocol (#13-CC-0110) and informed written consent and assent were obtained for all participants.

### Procedures

Participants walked on the treadmill (Bertec TM-06-B, Columbus, OH, United States) at a self-selected speed. Treadmill speed was initially estimated based on average pelvic velocity during overground walking and was adjusted while participants walked on the treadmill until each participant selected a comfortable walking speed at which walking was performed for 5 min of data collection.

Muscle activation was recorded using a wireless EMG system (Trigno, Delsys, Boston, MA, United States) at 1000 Hz. Surface electrodes were attached bilaterally to the tibialis anterior (TA), extensor hallucis longus (EH), lateral gastrocnemius (LG), soleus (SO), rectus femoris (RF), vastus lateralis (VL), semitendinosus (ST), and biceps femoris (BF) muscles (Perotto, 2011). Placement of EMG electrodes was per SENIAM guidelines (Hermens et al., 1999) and verified by manual muscle testing to ensure proper EMG signals. Kinematic data synchronized with EMG were collected at 100 Hz using ten motion capture cameras (Vicon, Lake Forest, CA, United States) and analyzed using Visual3D (C-Motion, Germantown, MD, United States) to detect gait events to calculate joint angles and to present synchronized EMG activation patterns across the gait cycle. EMG and kinematic data were collected and analyzed for the entire 5 min, treadmill walking data collection period.

### Muscle Synergy Extraction

Electromyography data were high-pass filtered (3rd order Butterworth) at 35 Hz, full-wave rectified, and low-pass filtered (3rd order Butterworth) at 6 Hz. For muscle synergy extraction, EMG data were split into windows of 20 gait cycles based on the heel contact, with 19 gait cycles of overlap between successive windows because we focused on relative contributions of each muscle and EMG normalization within each window. Also, a previous study demonstrated that reconstruction quality of muscle synergies was the highest for 20 gait cycles (Oliveira et al., 2014). We set the moving time window between 20 gait cycles windows to one gait cycle to maximize the chances of capturing a specific synergy that may only occur during a small (<20 gait cycles) period. After splitting, the data sets were time-interpolated to 2000 points and normalized by the maximum activation value within each window. Therefore, the EMG data set consisted of muscle × time matrices (EMGo) ranging from 0 to 1.

Non-negative matrix factorization was used to extract muscle synergies from EMGo (Lee and Seung, 1999). This linear decomposition technique computed muscle synergies according to the following formula:

$$E\,M\,G_{0}=\sum_{i=1}^{n}{\text{W}}_{\text{i}}\,{\text{C}}_{\text{i}}+e,E\,M\,G_{r}=\sum_{i=1}^{n}{\text{W}}_{\text{i}}\,{\text{C}}_{\text{i}}$$

where *n* is the number of muscle synergies ranging from 1 to 16, *i* is an identification number of each muscle synergy, W is a synergy structure (muscle × *n*) indicating weighting values of individual muscles for each synergy, C is a synergy activation (*n* × time) indicating time-varying synergy activation profiles, and *e* is residual error. EMGr is a reconstructed EMG matrix (muscle × time) resulting from the multiplication of W and C. To determine proper level of muscle synergies, we calculated the variability accounted for (VAF) as follows:

$$V\,A\,F=1-{\left(E\,M\,G_{o}-E\,M\,G_{r}\right)}^{2}/E\,M\,G_{o}^{2}$$

In this study, a VAF threshold was set at 90% in line with previous studies (Bowden et al., 2010; Clark et al., 2010; Dominici et al., 2011; Steele et al., 2015). Thus, we selected a minimal n that VAF was higher than 90%.

### Clustering Analysis

Using k-means clustering, similar muscle synergy structures were assigned into the same cluster. The size of the data matrix for clustering was 16 muscles × nW, where nW is the number of W matrices for all subjects (the number of time windows × subjects × synergy numbers). An inherent risk of the clustering process is that the presence of large numbers of samples near the cluster boundaries can lead to changes in clustering results if the analysis is run multiple times. To prevent inaccurate clustering, we used an iterative process to select the *k* value. This process was developed to find the minimal number of clusters that can be identified using a reliability test frequently conducted in clinical studies. Specifically, in an initial clustering process, the value of k was set at the minimum number of synergies in the time windows of 20 gait cycles among all subjects. Then, a discriminant analysis was used to revise the cluster assignment if necessary. In this supervised learning process, each synergy structure matrix and its cluster assignment were used to optimize the separation between clusters by projecting the data into a subspace that maximized the variance between means of projected clusters and minimized the variance within each cluster (Dixon and Brereton, 2009). The discriminant method was determined by the equality of cluster covariance matrices assessed using the Box’s *M* test (Box, 1949). If covariance matrices were equal, linear discriminant analysis (LDA) was used, otherwise quadratic discriminant analysis (QDA) was used (Dixon and Brereton, 2009). After discriminant analysis, we examined whether synergy structures extracted from the same time window were assigned into different clusters based on the requirement that non-negative matrix factorization extracts different or independent muscle synergies from EMGo. If the requisite condition was not satisfied, the clustering was repeated with a sequential increase in k. Next, the intra-class correlation coefficient (ICC) was used to test the similarity of the synergy structures assigned to each cluster by the discriminant analysis. To ensure accurate *k* value, this process was repeated 500 times. Experientially, we observed a stable *k*-value in prior studies at 100 iterations. Therefore, to build in a safety factor, we repeated the process 500 times to determine the correct *k* value. Then, we selected the case showing the most frequent *k* value for the repetition and the highest mean ICC value across individual clusters.

### Identifying Group-Specific Clusters

For each cluster, we computed the proportion of synergies originating from the TD or CP group to define whether the cluster is specific to one of the groups. To compare the proportions of the two groups within a cluster, we performed the two-proportion *z*-test (Sheskin et al., 2003) as follows:

$${\hat{p}}_{1}=x_{1}/n_{1},{\hat{p}}_{2}=x_{2}/n_{2},\hat{p}=x_{1}+x_{2}/n_{1}+n_{2},$$

$$z=\left[\left({\hat{p}}_{1}-{\hat{p}}_{2}\right)\right]/\sqrt{\hat{p}\,\left(1-\hat{p}\right)\,\left(1/n_{1}+1/n_{2}\right)}$$

where, each of *x*_*1*_ and *x*_*2*_ are the number of muscle synergies of CP and TD groups within each cluster and *n*_*1*_ and *n*_*2*_ are the total number of muscle synergies in the CP and TD groups, respectively. If the value of the z-score was higher than 1.96 or lower than −1.96, this cluster was defined as a CP-specific cluster or a TD-specific cluster (*p* < 0.05), respectively. If the value of the z-score was between −1.96 and 1.96, it was defined as a non-specific cluster. Based on the two-proportion *z*-test, each cluster was labeled as C, T, or N for CP-specific, TD-specific, or non-specific clusters, respectively. Then, within each cluster group, cluster ID was determined according to the rank of *z* values. For example, C1 indicated the CP-specific cluster showing the highest *z* value, indicating the most frequently observed muscle synergy in children with CP.

The cluster z-score is therefore a quantitative indicator of the constituent groups within it. For example, a high cluster z-score indicates that more children with CP use the muscle synergies assigned to it. Similarly, the number of extracted synergies from an individual participant assigned to any given cluster is an indicator of its prevalence during walking; that is, if an individual has numerous synergies assigned to a CP-specific cluster a higher proportion of their overall synergies are categorized as CP-specific. Thus, we quantified the group-specific level of muscle synergies for each participant through a weight-averaged z-score as follows:

$$w\,e\,i\,g\,h\,t-a\,v\,e\,r\,a\,g\,e\,d\,z-s\,c\,o\,r\,e=\sum_{i=1}^{n}z_{i}\times \left(n\,S\,y\,n_{i}/n_{g\,a\,i\,t\,\_\,c\,y\,c\,l\,e}\right)/n$$

where, i is the ith cluster identified from a participant. n is the number of clusters for a participant, z_*i*_ is the z-score of the ith cluster, nSyn_*i*_ is the participant’s number of muscle synergies within the ith cluster, and n_*gait_cycle*_ is the number of analyzed gait cycles of a participant. Similar to a cluster z-score, a participant who has a greater weight-averaged z-score indicates that his/her muscle synergies are CP-specific, and the opposite is TD-specific.

### Gait Performance Analysis

To investigate the effect of CP-specific muscle synergies on gait performance, we measured self-selected treadmill speed, step length asymmetry, step cadence asymmetry, and COM displacement of the pelvis. We also calculated the Gait Deviation Index on all with CP, with the mean TD group data used as the reference value. The asymmetry level of step length and cadence was calculated as follows (Reisman et al., 2013):

$$A\,s\,y\,m\,m\,e\,t\,r\,y\,l\,e\,v\,e\,l=\left|\left(V\,A\,L_{p\,a\,r\,e\,t\,i\,c}-V\,A\,L_{n\,o\,n\,p\,a\,r\,e\,t\,i\,c}\right)/\left(V\,A\,L_{p\,a\,r\,e\,t\,i\,c}+V\,A\,L_{n\,o\,n\,p\,a\,r\,e\,t\,i\,c}\right)\right|$$

where VALparetic and VALnon-paretic refer to the values of paretic and non-paretic step lengths or cadences, respectively. A larger value indicates a greater asymmetry of step length or cadence between legs. COM displacement of the pelvis was analyzed to quantify gait stability. In the study, ML-COM, AP-COM, and V-COM displacements were defined as the peak-to-peak distance of COM in mediolateral, anteroposterior, and vertical directions within each gait cycle, respectively. Each COM variables were averaged across gait cycles for analysis.

To examine the level of gait pathology in children with CP, in addition to GMFCS, we calculated the GDI (Schwartz and Rozumalski, 2008). For the GDI calculation, we used kinematic data in the TD group as the control data. The GDI score is calculated for each person from the following kinematic variables: pelvis (tilt, oblique, and rotation), hip (flexion, extension, abduction, adduction, internal rotation, and external rotation), knee (flexion and extension), ankle (dorsiflexion and plantar flexion), and foot (progression). The GDI score is ranged from 0 to 100 with 100 representing the TD group mean. Every 10-point reduction from 100 means 1 standard deviation away from the average of the TD group. GDI scores were calculated for dominant and non-dominant legs, respectively.

### Statistical Analysis

We performed statistical analysis using the independent *t*-test to compare the number of gait cycles, the mean number of synergies, self-selected treadmill speed, step length asymmetry, step cadence asymmetry, and COM displacement of the pelvis between two groups. To identify whether the group-specific level of muscle synergies in CP was correlated with gait performance, Spearman’s rank correlation coefficient (r) was calculated between the weight-averaged z-score and gait performance variables such as self-selected treadmill speed, step length asymmetry, step cadence asymmetry, and COM displacement of pelvis. The correlation was considered fair, moderate, or strong if *r* value was 0.25∼0.5, 0.5∼0.75, or 0.75∼1, respectively (Portney and Watkins, 2009). Moreover, we identified whether the weight-averaged z-score is correlated with GMFCS and GDI scores for CP cohort. The statistical significance was set at *p* < 0.05.

## Results

The number of analyzed gait cycles was not significantly different between groups (CP: 212.7 ± 53.2, TD: 213.4 ± 43.6, *t* = −0.032, *p* = 0.975). The mean number of synergies extracted from EMGo data set was slightly and significantly higher (*t* = −3.243, *p* = 0.009) in the TD group (3.96 ± 0.12, range: 3.62–4.00) compared to CP (3.45 ± 0.48, range: 2.98–4.00). The mean total number of synergies extracted across all strides for each individual was 656.6 ± 153.9 (range: 292–855) in CP group and 762.3 ± 159.1 (range: 368–935) in TD.

**FIGURE 1:**
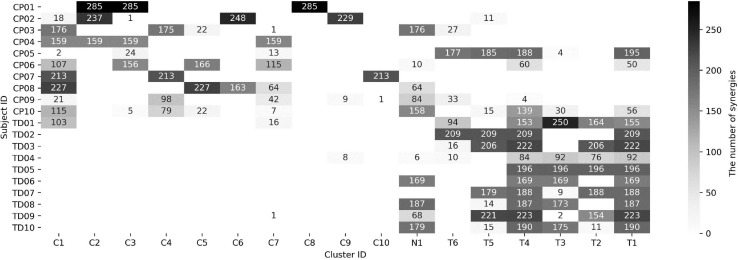
Muscle synergy assignment in 17 clusters. CP and TD indicate children with cerebral palsy and typical development, respectively. The brightness in each cell means the number of synergies assigned in a cluster (refer to right column). Each cell for CP-, TD-, and non-specific clusters was labeled as C, T, and N, respectively.

**FIGURE 2:**
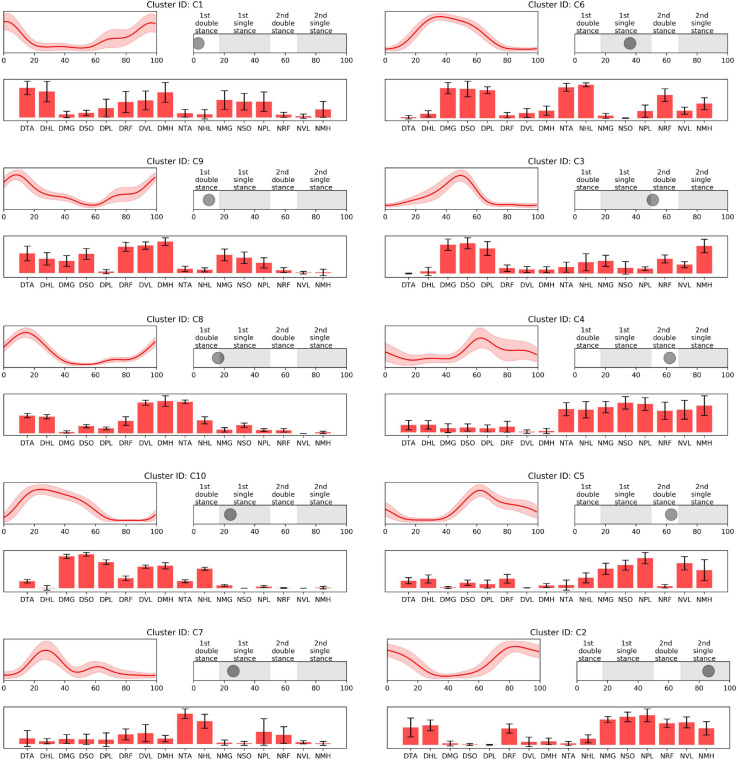
CP-specific synergy activations (top left), structures (low), and peak activation time in the gait cycle (top right). Cluster ID is displayed above each subfigure. Synergy activations and structures are expressed as mean and standard deviation. Subfigures are arranged based on peak activation time. The labels of bar plots are combined with leg side and muscle names as follows: D, dominant leg; N, non-dominant leg; TA, tibialis anterior; HL, extensor hallucis longus; MG, medial gastrocnemius; SO, soleus; PL, peroneus longus; RF, rectus femoris; VL, vastus lateralis; MH, medial hamstring.

**FIGURE 3:**
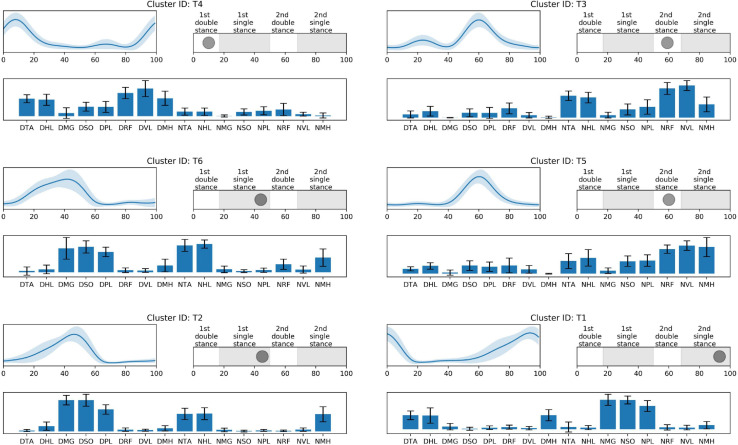
TD-specific synergy activations **(top left)**, structures **(low)**, and peak activation time in the gait cycle **(top right)**. Cluster ID is displayed above each subfigure. Synergy activations and structures are expressed as mean and standard deviation. Subfigures are arranged based on peak activation time. The labels of synergy structure are same with Figure 2.

**FIGURE 4:**
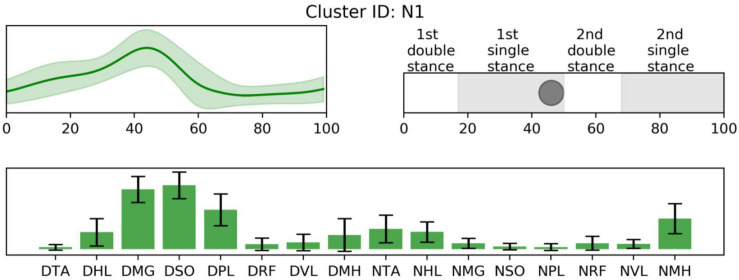
Non-specific synergy activation **(top left)**, structure **(low)**, and peak activation time in the gait cycle **(top right)**. Cluster ID is displayed above. Synergy activations and structures are expressed as mean and standard deviation. The labels of synergy structure are same with Figure 2.

In the iterative clustering, 17 distinct clusters were found across all subjects indicating that 17 unique muscle synergy structures were deployed during treadmill walking in our group of CP and TD participants (Figure 1). Among the 17 clusters, we found 10 CP-specific clusters (Figure 2). Among them, seven clusters (C2, 3, 4, 5, 6, 8, and 10) were observed only in children with CP. The other three clusters (C1, 7, and 9) were mostly observed in children with CP but were observed infrequently in the TD group. C8, 9, and 10 were predominantly observed in CP1, 2, and 7, respectively, indicating that these may be subject specific. Furthermore, we found six TD-specific clusters (Figure 3). Most TD-specific clusters (T1, 3, 4, 5, and 6) were also found in some children with CP, except for T2. Muscle synergies in T2 were observed only in the TD group. One non-specific cluster (N1) was observed in multiple participants from the TD and CP groups (Figure 4).

Prior to 16% of the gait cycle, C1, C8, C9 (Figure 2), and T4 (Figure 3) emerged, corresponding to initial contact and loading response of the dominant limb. In this period, the TD group co-activated dominant ankle dorsiflexor (TA and HL) and thigh muscles (RF, VL, and MH) as shown in T4. Whereas synergies structures were added with non-dominant ankle plantar flexors (C1: MG, SO, and PL) or dominant one (C9). In C8, non-dominant TA was co-activated with dominant dorsiflexor and thigh muscles (VL and MH). Between 24 and 51% of the gait cycle, the CP group had four clusters with peak activation C3, 6, 7, and 10 (Figure 2) and TD had T2 and T6 (Figure 3). Muscle synergies in T6 showed higher co-activation of both dominant plantar and non-dominant dorsiflexor muscles than those of T2. Synergy structures of C6 and C3 were similar to those of TD, non-dominant RF and MH were additionally co-activated, respectively. C10 was quite atypical, consisting of dominant plantar flexor, thigh (VL and MH), and non-dominant HL muscles. C7 exhibited co-activation of non-dominant dorsiflexors only. The one non-specific muscle synergy (N1) also showed peak activation in this period (Figure 4). This cluster mainly consisted of dominant plantar flexor activity. Between 51 and 63% of the gait cycle, T3 and T5 (Figure 3) and those of C4 and C5 (Figure 2) emerged. T3 and T5 consisted of non-dominant dorsiflexor and thigh muscles (T3: RF and VL, T5: RF, VL, and MH), symmetric with T4. In C4, all non-dominant muscles were co-activated. Also, its synergy activation varied between gait cycles (Figure 2). In C5, non-dominant thigh muscles (VL and MH) were co-activated with non-dominant plantar flexors instead of dorsiflexors. Between 86 and 93% of the gait cycle, we observed peak activation of T1 (Figure 3) and C2 (Figure 2). T1 contained non-dominant plantar flexors and dominant dorsiflexors, symmetric with T2. In case of C2, there was additional co-activation of dominant RF and non-dominant thigh muscles when compared with T1.

**FIGURE 5:**
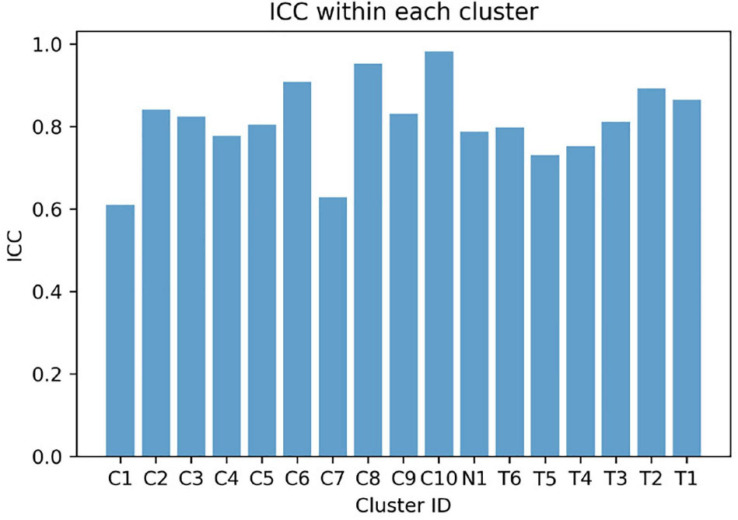
Intraclass correlation coefficient (ICC) values of muscle synergy structures within each cluster.

The mean weight-averaged z-score was 10.8 ± 10.6 and −17.3 ± 5.1 in the CP and TD groups, respectively. The weight-averaged z-score indicates whether a subject’s synergies are predominantly present in clusters specific to CP or TD. A higher positive weight averaged z-score indicates that muscle synergies are more CP-specific, and one in the opposite direction is more TD-specific. Since most synergies of children with TD were assigned into the TD-specific clusters, their weight-averaged z-score showed negative values in all participants (range: −9.5 ∼−27.7) while the mean scores in children with CP were mostly positive (range: −8.4 ∼ 24.1). Interestingly, two participants in the CP group showed negative values (CP05: −8.4 and CP10: −0.4) because a large number of synergies were assigned to the TD-specific clusters (Figure 1) whereas no participants from the TD group showed positive weight-averaged z-scores. The mean ICC value of muscle synergy structures across the 17 clusters were 0.81 ± 0.10, with a range of 0.61–0.98 (Figure 5).

**FIGURE 6:**
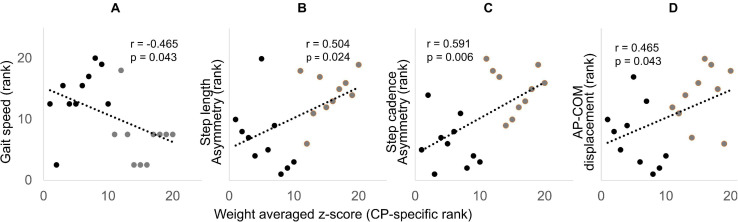
Spearman’s rank correlation scatter plots showing significant correlation between the weight-averaged z-score (*x*-axis) and self-selected treadmill speed **(A)**, step length asymmetry **(B)**, step cadence asymmetry **(C)**, and AP-COM **(D)**. The *x* and *y* axis are displayed in rank (descending order), with rank 1 being the highest. CP and TD data are presented with black and gray dots, respectively.

Gait performance variables such as treadmill speed, step length asymmetry, step cadence asymmetry, and AP-COM were significantly different between groups except for ML-COM and V-COM (Table 1). The Spearman’s rank correlation coefficient was significant between the weight-averaged z-scores and gait performance variables such as treadmill speed, step length asymmetry, step cadence asymmetry, and AP-COM displacement of the pelvis (Figure 6). Among them, the weight-averaged z-score showed moderate correlation with step length asymmetry (*r* = 0.504, *p* = 0.024) and step cadence asymmetry (*r* = 0.591, *p* = 0.006), and it was fair correlation with treadmill speed (*r* = −0.465, *p* = 0.043) and AP-COM displacement of the pelvis (*r* = 0.465, *p* = 0.043). GDI scores for dominant and non-dominant legs were 86.8 ± 9.2 and 83.5 ± 10.2, respectively, indicating that our CP cohort ranged 1–2 standard deviations from the TD group. GMFCS (*r* = −0.213, *p* = 0.554) and GDI scores (dominant leg: *r* = −0.055, *p* = 0.881; non-dominant leg: *r* = 0.188, *p* = 0.603) were not significantly correlated with the weight-averaged z-scores.

**TABLE 1:** Gait performance variables.

	**Self-selected treadmill speed**	**Step length asymmetry**	**Step cadence asymmetry**	**ML-COM**	**AP-COM**	**V-COM**
CP	0.80 ± 0.22	0.10 ± 0.13	0.06 ± 0.04	0.07 ± 0.02	0.06 ± 0.03	0.04 ± 0.01
TD	1.00 ± 0.12	0.01 ± 0.01	0.01 ± 0.01	0.06 ± 0.01	0.04 ± 0.01	0.03 ± 0.01
*p*	0.043*	0.024*	0.006*	0.523	0.043*	0.369

*: significant difference between groups (*p* < 0.05). CP, cerebral palsy group; TD, typically developing group; ML-COM, mediolateral center of mass displacement; AP-COM, anteroposterior center of mass displacement; V-COM, vertical center of mass displacement.

## Discussion

We identified 17 different muscle synergies (clusters) across our group of CP and TD participants; 16 of which were group-specific and one non-specific. A majority of the CP-specific clusters (7/10) consisted of muscle synergies extracted exclusively from children with CP and the remaining three CP-specific clusters were primarily seen in just one participant with TD. Thus, our results demonstrate that even mildly affected children with CP utilize distinct muscle synergies rarely observed in those with TD. The analysis protocol here combined machine learning and clustering algorithms to identify muscle synergies for walking specifically exhibited in children with unilateral CP. We recognize that cluster numbers and/or structures may vary depending on the sample and the specific cluster results here may not be generalizable to other individuals with CP; however, this would likely be the case regardless of how large the sample was. The major contribution here is an effective and quantitative approach to differentiate synergies between two groups at both a group and individual level, the latter of which could inform therapeutic prescription. An alternative approach would be to cluster within groups and then compare the clusters for those with typical development, which are likely to be more stable and predictable, to those for the group or individuals with CP. We believe that method would be far less quantitative and precise. Our method could be used for differentiating typical gait from any clinical population and serves to reveal unique patterns that emerge as one recovers from (or develops with) a brain injury. The finding of some more common patterns that emerged in this group of children with unilateral and predominantly mild CP extend the current knowledge on the characteristics of muscle synergies in CP.

This study also reinforces previous reports that altered cortical and spinal motor neural networks in children with CP produce specific muscle synergies that are not observed in healthy controls (Myklebust, 1990; Eyre, 2007; Friel and Martin, 2007). The distribution of CP-specific vs. TD-specific synergies within an individual’s repertoire was variable in our CP cohort: 4/10 deployed only CP-specific muscle synergies whereas 6/10 deployed both CP- and TD-specific synergies (Figure 1). Interestingly, only 1/10 TD participants deployed CP-specific synergies in an appreciable number of strides. Furthermore, of the group specific clusters, seven were observed only in CP whereas only one was exclusive to TD. Taken together, these findings suggest that children with CP utilize unique and individual-specific patterns of motor control during walking compared to children with TD. Previous studies reported that postnatal development changes the organization of spinal interneurons by the activation of the corticospinal tract (Eyre et al., 2001; Friel and Martin, 2005; Chakrabarty et al., 2009). Initially, the axons of the corticospinal tract terminate on the spinal motor neurons bilaterally (Eyre et al., 2001; Friel and Martin, 2007). As development proceeds, some contralateral and most ipsilateral terminations are pruned based on the activation of the corticospinal tract (Li and Martin, 2000). However, brain injuries during the development process may result in fundamentally disordered spinal cord circuitry such as reciprocal excitation of antagonistic muscles (Myklebust, 1990) and simultaneous bilateral motor responses (Eyre, 2007). These data clearly show that children with CP generally exhibited greater co-activation of muscles, or alternately stated, less selectivity, when comparing synergy structures with those with TD. The co-activation of antagonist leg muscles in CP has been well-established (Berger et al., 1982) and is attributed to decreased cortical control for descending motor pathways in an injured hemisphere (Cao et al., 1994) and increased overlap of sensorimotor cortical activities (Burton et al., 2009).

Participants with CP presented synergy structures such as C2, 3, 4, 5, 6, 8, and 10, which were not present in any children with TD. Also, C1, 7, and 9 were predominant in the CP population. Based on the peak activation time of the muscle synergy in the walking cycle, CP-specific clusters are arranged in order C1, 9, 8, 10, 7, 6, 3, 4, 5, and 2 (Figure 2). Among them, C1, 9, and 8 were responsible for dominant heel contact and loading response in the double leg stance. In this period, children with TD co-activated dominant ankle dorsiflexor and knee extensor muscles only as shown in T4, while those with CP additionally co-activated dominant or non-dominant ankle plantar flexors or non-dominant ankle plantar flexors. The additional muscle co-activation in the CP-specific clusters has been considered in some cases as the merging of muscle synergies observed in healthy controls (Clark et al., 2010; Cheung et al., 2012). For instance, synergy structures of C1 show the merging of those of T1 and T4. In case of C9, its synergy structure presents co-activation of both ankle dorsiflexors and plantar flexors. This pattern can be interpreted as antagonist co-activation, the characteristic of locomotion in children with CP (Damiano et al., 2000; Cappellini et al., 2016), which cannot be judged as the merging of muscle synergies in healthy controls. We also found a new pattern that is not well explained as either a merged synergy or antagonist co-activation. For instance, the synergy structure of C8 showed additional co-activation of non-dominant TA muscle. Yet, since the synergy structure of C8 emerged only in one participant, CP1, this specific synergy pattern may indicate a subject-specific or at least less frequent strategy.

In single-leg stance on the dominant leg, we observed various group-specific features of muscle synergy. Specifically, children with TD utilized T6 or T2 patterns, consisting of dominant ankle plantar flexors and non-dominant ankle dorsiflexors. These patterns correspond to the ability to push the body forward through the dominant ankle flexor muscles and lift the non-dominant legs. However, in case of CP-specific clusters, as shown in the clusters for the loading response, the synergy structure of C6 showed additional co-activation of non-dominant thigh muscles. In this period, other specific patterns were also observed. For instance, C3 exhibited decreased activation in non-dominant ankle dorsiflexors. Given the synergy structure of C3 and C6, activation instead of non-dominant RF or MH may be the adaptive compensation for poor selective control in that muscle. Unlike the case of C8, these patterns were observed in several children with CP. This finding supports the conclusion that there are some common or at least more frequent muscle synergies that are shared by those with the diagnosis of unilateral CP. In contrast to C3 and C6, C7 included the activation of non-dominant ankle dorsiflexors, without dominant leg muscle activation. This pattern resembles the fractionation of muscle synergies observed in stroke survivors (Cheung et al., 2012). C10 showed a somewhat complex pattern. Children with CP having this pattern additionally co-activated dominant thigh muscles. Moreover, this pattern did not include co-activation of non-dominant ankle dorsiflexors, TA. Instead, non-dominant HL, great toe extensor, and ankle dorsiflexor, were co-activated with other muscles.

Furthermore, in single-leg stance on the dominant leg, participants showed the most variable synergy patterns. In this phase, there was also a non-specific synergy pattern, N1. One interpretation of the variable clusters in dominant limb single leg stance is that there is the potential for accessing a larger repertoire at this point in the gait cycle. For this phase, dominant ankle plantar flexors provide forward propulsion of the body and non-dominant flexors lift the ipsilateral leg. Because dominant leg muscles should be largely unimpaired in our CP group, to prepare for weight-bearing of the non-dominant leg, they may utilize their inherent motor strategies in various ways that may be different from those in TD. This notion concurs with the concept of motor abundance that the central nervous system utilizes multiple variations of muscle coordination to achieve a given motor task (Gera et al., 2010). Previous studies also showed that the injured brain utilizes more variable motor patterns using residual capacity (Sanger, 2006; Kim et al., 2016, 2018). However, after the loading response of the non-dominant leg, the number of CP-specific clusters notably decreased. This finding indicates decreased motor capability to produce various patterns regarding adaptive compensation (Levin et al., 2009; Kim et al., 2017). We postulate that the upper motor neuron dysfunction (weakness, dystonia, hyperreflexia, spasticity, and increased antagonist co-contraction) manifested in the non-dominant leg of the CP group restricts the repertoire of compensatory motor strategies available in that limb.

Around 60% of the gait cycle, corresponding to the loading response of the non-dominant leg, children with TD utilized T3 or T5 patterns while those with CP exhibited C4 or C5. T3 and T5 are co-activation of the non-dominant thigh and dorsiflexion muscles and resemble T4, co-activation of contralateral muscles. Interestingly, in this phase, CP-specific clusters showed totally different patterns when compared with TD-specific clusters. C4 represents the most unique features of the CP-specific clusters observed in the study, co-activating all non-dominant leg muscles. This pattern has been considered as causing spastic gait in children with CP (Crenna, 1998) and interrupting their selective voluntary motor control (Fowler et al., 2009). In case of C5, non-dominant ankle plantar flexors were co-activated with thigh muscles while the ankle dorsiflexors and thigh muscles of the same side were co-activated for initial contact in TD-specific clusters, T3 and T5. In a single-leg stance on the non-dominant leg, T1 emerged and was symmetric with T2. However, as with the loading phase of the non-dominant leg, a CP-specific cluster in this phase, C2, exhibited a unique pattern. In C2, there was co-activation of non-dominant thigh muscles in addition to T1.

We utilized the weight-averaged z-score to quantify the relative percentage of muscle synergies expressed by an individual participant that were group-specific. A greater magnitude of the weight-averaged z-score means that a greater percentage of an individual’s muscle synergies are group specific, while the sign indicates the group, i.e., positive scores are CP-specific and negative values are TD-specific. Our findings showed that both CP and TD groups had magnitudes beyond 1.96, indicating that each primarily deployed synergies specific to their own group. Yet, the magnitude of the weight-averaged z-score was greater in the TD group when compared with the CP group, which indicates that very few children with TD deployed synergies observed in those with CP whereas several children with CP deployed synergies observed in TD. This suggests that at least some children with unilateral CP develop and retain typical patterns of muscle activations. But, the standard deviation of the score was greater within children with CP, supporting that their synergies are more variable between individuals than those of TD (Kim et al., 2018). Furthermore, our results demonstrate that the weight-averaged z-scores were correlated with gait performance variables. Specifically, an increase in the weight-averaged z-score correlated with a decrease in self-selected treadmill speed and an increase in the step length asymmetry, step cadence asymmetry, and AP-COM displacement of the pelvis. These results indicate that greater relative deployment of CP-specific muscle synergies underlies poorer gait performance in our cohort. Also, our findings more broadly demonstrate that the muscle synergy structures as well as numbers are associated with the performance level of a functional motor task.

Based on our findings, we propose the use of more individually tailored rehabilitation strategies for gait training based on individual muscle synergy patterns. Analyzing muscle synergies in individuals with brain injuries may provide new insights by visualizing their altered motor command. For example, based on our analysis, clinicians can understand that their patient uses some atypical muscle co-activation in a particular gait phase. Then, during rehabilitation training sessions, the clinician can focus on the atypical pattern and try to inhibit or facilitate specific muscle activations to resemble patterns seen in those without CP. In this study, we found that CP-specific clusters generally have four possible characteristics that may differentiate them from TD-specific clusters. The first characteristic is the merging of muscle synergies identified in TD-specific clusters, e.g., C1. The training goal would be to dissociate merged muscle synergies by trying to promote activation in muscles that best differentiate the synergies and relate to better functional performance. The second characteristic is strong antagonist co-activation such as C4. In this study, most individuals who utilize pattern C4 were classified as GMFCS level II. Spasticity reduction or intensive practice to train better selective control may be effective in rehabilitation of individuals who deploy this synergy pattern. The third characteristic is the loss of activation in a specific muscle (e.g., ankle dorsiflexors) as shown in C7. Here one should emphasize selective control training or strengthening of that muscle if possible. The fourth characteristic is a complex pattern like C10. In this case, rehabilitation training may need to combine multiple approaches mentioned above. However, the extent to which motor training for any of these specific impairments can produce positive and lasting effects is not still well known. We expect that muscle synergy analyses offer a more mechanistic approach for intervention at an individual level as well as a novel method to discriminate levels of function and to assess outcomes.

Limitations of this study are the relatively small number of children with CP, and that only the two most functional GMFCS levels were represented. These issues may have affected correlation coefficients in our study regarding GMFCS and GDI scores, and limited the ability to do within-group correlations. Despite significant correlations between overall gait function and deployment of CP-specific synergies, we also observed a range of gait performance in individuals who deployed mostly CP-specific synergies. Similar to a previous study that examined whether the number of muscle synergies is correlated with the clinical measurement of motor dysfunction (Bowden et al., 2010), a future study with a larger number of participants with CP with a broader range of functional levels is necessary to better address whether the weight-averaged z-score is correlated with functional gait performance in those with CP and other motor disabilities. Another limitation is that muscle activation was recorded mainly in lower limb flexors and extensors. Muscle synergies extracted from additional muscles that function in other planes would provide greater insights into the complex 3D gait abnormalities in CP. In the study, the muscle synergies were analyzed for treadmill walking. A previous study demonstrated that treadmill locomotion shares similar muscle synergy structures with overground locomotion (Oliveira et al., 2016; Mileti et al., 2020) although synergy activations were more consistent in the treadmill condition (Mileti et al., 2020). Since we classified group-specific synergies based on synergy structures, we assume that walking environment may rarely influence the results.

In conclusion, we found CP-specific muscle synergies that represent an altered repertoire of muscle coordination patterns during walking when compared with age-matched controls. CP-specific muscle synergies had four differentiating characteristics: (1) merging of two TD synergies, (2) excessive antagonist co-activation, (3) decreased muscle activation compared to TD, or (4) a complex or atypical pattern. Also, our results demonstrated that greater utilization of CP-specific muscle synergies was related to poorer gait performance. Our approach identifying subject-specific muscle synergy patterns may provide useful information to establish more individually tailored rehabilitation strategies to improve gait performance.

## Data Availability Statement

The data sets for this study are available upon request to the corresponding author.

## Ethics Statement

The studies involving human participants were reviewed and approved by the Institutional Review Board of NIH. Informed consent to participate in this study was provided by the participants’ legal guardian/next of kin.

## Author Contributions

YK, TB, and DD conceived and designed the research, interpreted results of the experiments, edited and revised the manuscript, and approved final version of manuscript. TB and YK performed the experiments. YK analyzed the data, prepared the figures, and drafted the manuscript.

## Conflict of Interest

The authors declare that the research was conducted in the absence of any commercial or financial relationships that could be construed as a potential conflict of interest.

## References

[B1] Allen J. L. McKay J. L. Sawers A. Hackney M. E. Ting L. H. (2017). Increased neuromuscular consistency in gait and balance after partnered, dance-based rehabilitation in parkinson’s disease. *J. Neurophysiol.* 118 363–373. 10.1152/jn.00813.2016 28381488 PMC5501921

[B2] Berger W. (1998). Characteristics of locomotor control in children with cerebral palsy. *Neurosci. Biobehav. Rev.* 22 579–582. 10.1016/S0149-7634(97)00047-X 9595572

[B3] Berger W. Quintern J. Dietz V. (1982). Pathophysiology of gait in children with cerebral palsy. *Electroencephalogr. Clin. Neurophysiol.* 53 538–548. 6177498 10.1016/0013-4694(82)90066-9

[B4] Bowden M. G. Clark D. J. Kautz S. A. (2010). Evaluation of abnormal synergy patterns poststroke: Relationship of the fugl-meyer assessment to hemiparetic locomotion. *Neurorehabil. Neural Repair* 24 328–337. 10.1177/1545968309343215 19794132 PMC4434590

[B5] Box G. E. P. (1949). A general distribution theory for a class of likelihood criteria. *Biometrika* 36 317–346. 10.2307/2332671 15402070

[B6] Brouwer B. Ashby P. (1991). Altered corticospinal projections to lower limb motoneurons in subjects with cerebral palsy. *Brain* 114 1395–1407. 10.1093/brain/114.3.1395 2065257

[B7] Burton H. Dixit S. Litkowski P. Wingert J. R. (2009). Functional connectivity for somatosensory and motor cortex in spastic diplegia. *Somatosens. Mot. Res.* 26 90–104. 10.3109/08990220903335742 20047510 PMC2804938

[B8] Cao Y. Vikingstad E. M. Huttenlocher P. R. Towle V. L. Levin D. N. (1994). Functional magnetic resonance studies of the reorganization of the human hand sensorimotor area after unilateral brain injury in the perinatal period. *Proc. Natl. Acad. Sci.* 91 9612–9616. 10.1073/pnas.91.20.9612 7937815 PMC44863

[B9] Cappellini G. Ivanenko Y. P. Martino G. MacLellan M. J. Sacco A. Morelli D. (2016). Immature spinal locomotor output in children with cerebral palsy. *Front. Physiol.* 7:1–21. 10.3389/fphys.2016.00478 27826251 PMC5078720

[B10] Chakrabarty S. Shulman B. Martin J. H. (2009). Activity-dependent codevelopment of the corticospinal system and target interneurons in the cervical spinal cord. *J. Neurosci.* 29 8816–8827. 10.1523/jneurosci.0735-09.2009 19587289 PMC3849701

[B11] Cheung V. C. K. Turolla A. Agostini M. Silvoni S. Bennis C. Kasi P. (2012). Muscle synergy patterns as physiological markers of motor cortical damage. *Proc. Natl. Acad. Sci.* 109 14652–14656. 10.1073/pnas.1212056109 22908288 PMC3437897

[B12] Clark D. J. Ting L. H. Zajac F. E. Neptune R. R. Kautz S. A. (2010). Merging of healthy motor modules predicts reduced locomotor performance and muscle coordination complexity post-stroke. *J. Neurophysiol.* 103 844–857. 10.1152/jn.00825.2009 20007501 PMC2822696

[B13] Crenna P. (1998). Spasticity and “spastic” gait in children with cerebral palsy. *Neurosci. Biobehav. Rev.* 46:S0149. 10.1016/S0149-7634(97)00046-8 9595571

[B14] Damiano D. L. Kelly L. E. Vaughn C. L. (1995). Effects of quadriceps femoris muscle strengthening on crouch gait in children with spastic diplegia. *Phys. Ther.* 75 658–667. 10.1093/ptj/75.8.658 7644570

[B15] Damiano D. L. Martellotta T. L. Sullivan D. J. Granata K. P. Abel M. F. (2000). Muscle force production and functional performance in spastic cerebral palsy: Relationship of cocontraction. *Arch. Phys. Med. Rehabil.* 81 895–900. 10.1053/apmr.2000.5579 10896001

[B16] Damiano D. L. Wingert J. R. Stanley C. J. Curatalo L. (2013). Contribution of hip joint proprioception to static and dynamic balance in cerebral palsy: a case control study. *J. Neuroeng. Rehabil.* 10:57. 10.1186/1743-0003-10-57 23767869 PMC3691826

[B17] Dixon S. J. Brereton R. G. (2009). Comparison of performance of five common classifiers represented as boundary methods: Euclidean Distance to Centroids, Linear Discriminant Analysis, Quadratic Discriminant Analysis, Learning Vector Quantization and Support Vector Machines, as dependent on. *Chemom. Intell. Lab. Syst.* 95 1–17. 10.1016/j.chemolab.2008.07.010

[B18] Dominici N. Dominici N. Ivanenko Y. P. Cappellini G. Avella A. Mondì V. (2011). Locomotor Primitives in Newborn Babies and Their Development. *Science* 997 997–999. 10.1126/science.1210617 22096202

[B19] Eyre J. A. (2007). Corticospinal tract development and its plasticity after perinatal injury. *Neurosci. Biobehav. Rev.* 31 1136–1149. 10.1016/j.neubiorev.2007.05.011 18053875

[B20] Eyre J. A. Taylor J. P. Villagra F. Smith M. Miller S. (2001). Evidence of activity-dependent withdrawal of corticospinal projections during human development. *Neurology* 57 1543–1554. 10.1212/wnl.57.9.1543 11706088

[B21] Fowler E. G. Staudt L. A. Greenberg M. B. Oppenheim W. L. (2009). Selective Control Assessment of the Lower Extremity (SCALE): development, validation, and interrater reliability of a clinical tool for patients with cerebral palsy. *Dev. Med. Child Neurol.* 51 607–614. 10.1111/j.1469-8749.2008.03186.x 19220390

[B22] Friel K. M. Martin J. H. (2005). Role of sensory−motor cortex activity in postnatal development of corticospinal axon terminals in the cat. *J. Comp. Neurol.* 485 43–56. 10.1002/cne.20483 15776437

[B23] Friel K. M. Martin J. H. (2007). Bilateral activity-dependent interactions in the developing corticospinal system. *J. Neurosci.* 27 11083–11090. 10.1523/jneurosci.2814-07.2007 17928450 PMC2740658

[B24] Gera G. Freitas S. Latash M. Monahan K. Schöner G. Scholz J. (2010). Motor abundance contributes to resolving multiple kinematic task constraints. *Motor Control.* 14 83–115. 10.1123/mcj.14.1.83 20237405 PMC2843002

[B25] Hermens H. J. Freriks B. Merletti R. Stegeman D. Blok J. Rau G. (1999). European recommendations for surface electromyography. *Roessingh. Res. Dev.* 8 13–54.

[B26] Ivanenko Y. P. Cappellini G. Dominici N. Poppele R. E. Lacquaniti F. (2005). Coordination of locomotion with voluntary movements in humans. *J. Neurosci.* 25 7238–7253. 10.1523/jneurosci.1327-05.2005 16079406 PMC6725226

[B27] Ivanenko Y. P. Grasso R. Zago M. Molinari M. Scivoletto G. Castellano V. (2003). Temporal components of the motor patterns expressed by the human spinal cord reflect foot kinematics. *J. Neurophysiol.* 90 3555–3565. 10.1152/jn.00223.2003 12853436

[B28] Ivanenko Y. P. Poppele R. E. Lacquaniti F. (2004). Five basic muscle activation patterns account for muscle activity during human locomotion. *J. Physiol.* 556 267–282. 10.1113/jphysiol.2003.057174 14724214 PMC1664897

[B29] Kim Y. Bulea T. C. Damiano D. L. (2018). Children with cerebral palsy have greater stride-to-stride variability of muscle synergies during gait than typically developing children: implications for motor control complexity. *Neurorehabil. Neural. Repair* 32 834–844. 10.1177/1545968318796333 30223739 PMC7271466

[B30] Kim Y. Kim W. S. Koh K. Yoon B. C. Damiano D. L. Shim J. K. (2016). Deficits in motor abilities for multi-finger force control in hemiparetic stroke survivors. *Exp. Brain Res.* 234 2391–2402. 10.1007/s00221-016-4644-2 27071926

[B31] Kim Y. Koh K. Yoon B. C. Kim W. S. Shin J. H. Park H. S. (2017). Examining impairment of adaptive compensation for stabilizing motor repetitions in stroke survivors. *Exp. Brain Res.* 235 3543–3552. 10.1007/s00221-017-5074-5 28879510

[B32] Lacquaniti F. Ivanenko Y. P. Zago M. (2012). Patterned control of human locomotion. *J. Physiol.* 590 2189–2199. 10.1113/jphysiol.2011.215137 22411012 PMC3424743

[B33] Lee D. D. Seung H. S. (1999). Learning the parts of objects by non-negative matrix factorization. *Nature* 401 788–791. 10.1038/44565 10548103

[B34] Leonard C. T. Hirschfeld H. Forssberg H. (2008). The development of independent walking in children with cerebral palsy. *Dev. Med. Child Neurol.* 33 567–577. 10.1111/j.1469-8749.1991.tb14926.x 1879620

[B35] Levin M. F. Kleim J. A. Wolf S. L. (2009). What do motor “recovery” and “compensation” mean in patients following stroke? *Neurorehabil. Neural. Repair* 23 313–319. 10.1177/1545968308328727 19118128

[B36] Li Q. Martin J. H. (2000). Postnatal development of differential projections from the caudal and rostral motor cortex subregions. *Exp. Brain Res.* 134 187–198. 10.1007/s002210000454 11037285

[B37] McGowan C. P. Neptune R. R. Clark D. J. Kautz S. A. (2010). Modular control of human walking: Adaptations to altered mechanical demands. *J. Biomech.* 43 412–419. 10.1016/j.jbiomech.2009.10.009 19879583 PMC2813323

[B38] Mileti I. Serra A. Wolf N. Munoz-Martel V. Ekizos A. Palermo E. (2020). Muscle activation patterns are more constrained and regular in treadmill than in overground human locomotion. *Front. Bioeng. Biotechnol.* 8:581619. 10.3389/fbioe.2020.581619 33195143 PMC7644811

[B39] Myklebust B. M. (1990). A review of myotatic reflexes and the development of motor control and gait in infants and children: a special communication. *Phys. Ther.* 70 188–203. 10.1093/ptj/70.3.188 2304976

[B40] Oliveira A. S. Gizzi L. Farina D. Kersting U. G. (2014). Motor modules of human locomotion: influence of EMG averaging, concatenation, and number of step cycles. *Front. Hum. Neurosci.* 8:1–9. 10.3389/fnhum.2014.00335 24904375 PMC4033063

[B41] Oliveira A. S. Gizzi L. Ketabi S. Farina D. Kersting U. G. (2016). Modular control of treadmill vs overground running. *PLoS One* 11:e0153307. 10.1371/journal.pone.0153307 27064978 PMC4827843

[B42] Perotto A. O. (2011). *Anatomical guide for the electromyographer: the limbs and trunk.* London: Charles C Thomas Publisher.

[B43] Portney L. G. Watkins M. P. (2009). *Foundations of clinical research: Applications to practice.* Upper Saddle River, NJ: Pearson/Prentice Hall.

[B44] Prosser L. A. Lauer R. T. VanSant A. F. Barbe M. F. Lee S. C. K. (2010). Variability and symmetry of gait in early walkers with and without bilateral cerebral palsy. *Gait Posture* 31 522–526. 10.1016/j.gaitpost.2010.03.001 20338763 PMC2862475

[B45] Reisman D. S. McLean H. Keller J. Danks K. A. Bastian A. J. (2013). Repeated split-belt treadmill training improves poststroke step length asymmetry. *Neurorehabil. Neural. Repair* 27 460–468. 10.1177/1545968312474118 23392918 PMC3738184

[B46] Rodriguez K. L. Roemmich R. T. Cam B. Fregly B. J. Hass C. J. (2013). Persons with Parkinson’s disease exhibit decreased neuromuscular complexity during gait. *Clin. Neurophysiol.* 124 1390–1397. 10.1016/j.clinph.2013.02.006 23474055 PMC3679225

[B47] Romkes J. Brunner R. (2007). An electromyographic analysis of obligatory (hemiplegic cerebral palsy) and voluntary (normal) unilateral toe-walking. *Gait Posture* 26 577–586. 10.1016/j.gaitpost.2006.12.010 17275305

[B48] Sanger T. D. (2006). Arm trajectories in dyskinetic cerebral palsy have increased random variability. *J. Child Neurol.* 21 551–557. 10.1177/08830738060210070201 16970842

[B49] Schwartz M. H. Rozumalski A. (2008). The Gait Deviation Index: a new comprehensive index of gait pathology. *Gait Posture* 28 351–357. 10.1016/j.gaitpost.2008.05.001 18565753

[B50] Sheskin D. J. Kafadar K. Sheskin D. J. (2003). *Handbook of parametric and nonparametric statistical procedures.* United Kingdom: Chapman and Hall/CRC.

[B51] Shuman B. R. Goudriaan M. Desloovere K. Schwartz M. H. Steele K. M. (2018). Associations Between Muscle Synergies and Treatment Outcomes in Cerebral Palsy Are Robust Across Clinical Centers. *Arch. Phys. Med. Rehabil.* 99 2175–2182. 10.1016/j.apmr.2018.03.006 29649451 PMC6179956

[B52] Steele K. M. Munger M. E. Peters K. M. Shuman B. R. Schwartz M. H. (2019). Repeatability of electromyography recordings and muscle synergies during gait among children with cerebral palsy. *Gait Posture* 67 290–295. 10.1016/j.gaitpost.2018.10.009 30396059 PMC6283402

[B53] Steele K. M. Rozumalski A. Schwartz M. H. (2015). Muscle synergies and complexity of neuromuscular control during gait in cerebral palsy. *Dev. Med. Child Neurol.* 57 1176–1182. 10.1111/dmcn.12826 26084733 PMC4683117

[B54] Ting L. H. Chiel H. J. Trumbower R. D. Allen J. L. McKay J. L. Hackney M. E. (2015). Neuromechanical principles underlying movement modularity and their implications for rehabilitation. *Neuron* 86 38–54. 10.1016/j.neuron.2015.02.042 25856485 PMC4392340

[B55] Torres-Oviedo G. Ting L. H. (2010). Subject-specific muscle synergies in human balance control are consistent across different biomechanical contexts. *J. Neurophysiol.* 103 3084–3098. 10.1152/jn.00960.2009 20393070 PMC2888239

[B56] Zollinger M. Degache F. Currat G. Pochon L. Peyrot N. Newman C. J. (2016). External mechanical work and pendular energy transduction of overground and treadmill walking in adolescents with unilateral cerebral palsy. *Front. Physiol.* 7:121. 10.3389/fphys.2016.00121 27148062 PMC4829600

